# High-density 80 K SNP array is a powerful tool for genotyping *G. hirsutum* accessions and genome analysis

**DOI:** 10.1186/s12864-017-4062-2

**Published:** 2017-08-23

**Authors:** Caiping Cai, Guozhong Zhu, Tianzhen Zhang, Wangzhen Guo

**Affiliations:** 1 State Key Laboratory of Crop Genetics & Germplasm Enhancement, Hybrid Cotton R & D Engineering Research Center, Ministry of Education, Nanjing Agricultural University, Nanjing, 210095 China; 2 State Key Laboratory of Crop Genetics & Germplasm Enhancement, Nanjing Agricultural University, Nanjing, 210095 Jiangsu Province People’s Republic of China

**Keywords:** Single nucleotide polymorphism (SNP), Array, Upland cotton, Genotyping identification, Genome-wide association studies (GWAS), Molecular breeding

## Abstract

**Background:**

High-throughput genotyping platforms play important roles in plant genomic studies. Cotton (*Gossypium* spp.) is the world’s important natural textile fiber and oil crop. Upland cotton accounts for more than 90% of the world’s cotton production, however, modern upland cotton cultivars have narrow genetic diversity. The amounts of genomic sequencing and re-sequencing data released make it possible to develop a high-quality single nucleotide polymorphism (SNP) array for intraspecific genotyping detection in cotton.

**Results:**

Here we report a high-throughput CottonSNP80K array and its utilization in genotyping detection in different cotton accessions. 82,259 SNP markers were selected from the re-sequencing data of 100 cotton cultivars and used to produce the array on the Illumina Infinium platform. 77,774 SNP loci (94.55%) were successfully synthesized on the array. Of them, 77,252 (99.33%) had call rates of >95% in 352 cotton accessions and 59,502 (76.51%) were polymorphic loci. Application tests using 22 cotton accessions with parent/F_1_ combinations or with similar genetic backgrounds showed that CottonSNP80K array had high genotyping accuracy, good repeatability, and wide applicability. Phylogenetic analysis of 312 cotton cultivars and landraces with wide geographical distribution showed that they could be classified into ten groups, irrelevant of their origins. We found that the different landraces were clustered in different subgroups, indicating that these landraces were major contributors to the development of different breeding populations of modern *G. hirsutum* cultivars in China. We integrated a total of 54,588 SNPs (MAFs >0.05) associated with 10 salt stress traits into 288 *G. hirsutum* accessions for genome-wide association studies (GWAS), and eight significant SNPs associated with three salt stress traits were detected.

**Conclusions:**

We developed CottonSNP80K array with high polymorphism to distinguish upland cotton accessions. Diverse application tests indicated that the CottonSNP80K play important roles in germplasm genotyping, variety verification, functional genomics studies, and molecular breeding in cotton.

**Electronic supplementary material:**

The online version of this article (doi:10.1186/s12864-017-4062-2) contains supplementary material, which is available to authorized users.

## Background

Cotton (*Gossypium* spp.) is the largest source of renewable fiber in the world and is also a significant oil crop. Upland cotton accounts for more than 90% of the world crop. However, modern upland cotton cultivars have narrow genetic diversity, due to their development from the limited quantity of resources in the United States [1]. Molecular markers have been successfully used in genotyping identification of cotton accessions and genomics-based cotton improvement. Among these, simple sequence repeat (SSR) markers are the most widely used type of molecular marker in cotton. So far, 19,010 SSR markers have been registered in CottonDB (http://cottondb.org/), and 100,290 microsatellites have been mined in the genome of *G. hirsutum*, where 77,996 SSR markers been developed [2]. However, SSR markers have disadvantages; for example, they are low-throughput, labor-intensive and time-consuming as a genotyping platform. In addition, the low density of SSR markers could cause the loss of linkages between markers and target trait loci/genes [3]. Hence, high-throughput molecular markers are imperative to saturate cotton genetic maps, and to significantly improve gene/quantitative trait loci (QTL) mapping for genomic studies and marker-assisted selection (MAS) breeding [4].

Single nucleotide polymorphisms (SNPs) are the most abundant DNA sequence variation present in plant genomes, with virtually unlimited, evenly distributed along the genome, bi-allelic and co-dominant characteristics. In cotton, several studies on genome-wide SNP marker development and utilization have been reported recently. Using BAC-end sequences of 12 *G. hirsutum* lines, one *G. barbadense* acc. 3–79 and one *G. longicalyx* line, genome-wide SNPs were mined. Of them, a total of 132,262 intraspecific SNPs have been developed in *G. hirsutum*, while 223,138 and 470,631 interspecific SNPs have been identified between *G. hirsutum* and *G. barbadense* or *G. longicalyx*, respectively [5]. By means of genotyping-by-sequencing (GBS) of 6071 SNPs and 223 SSRs, genome-wide association studies (GWAS) analysis was conducted using 547 recombination inbreed lines (RILs) of a multi-parent advanced generation inter-cross (MAGIC) population in four environments to identify genes/QTLs associated with fiber quality traits. As a result, a QTL cluster associated with four fiber quality traits, short fiber content (SFC), strength (STR), length (UHM) and uniformity (UI), was identified on A07, and a candidate gene, *GhRBB1_A07*, encoding a regeneration of bulb biogenesis 1 protein was further investigated [6]. Based on the specific-locus amplified fragment sequencing (SLAF-seq) of 355 upland cotton accessions, a total of 81,675 SNP markers was used for GWAS analysis of lint percentage [7] and early maturity [8]. Taken together, these findings suggest that SNPs play important roles in cotton genetics and breeding studies.

Compared to re-sequencing analysis, SNP arrays can produce large-scale genotyping data through one hybridization procedure at a relatively low cost. So far, there are nearly a hundred different genotyping techniques to meet different SNP array needs, with different sample sizes and numbers of SNP loci. In high-density SNP genotyping, Illumina (Infinium ® technology) and Affymetrix (Affymetrix GeneTitan ® technology) are two of the most widely used SNP genotyping platforms in a variety of species. In agricultural crops, a variety of SNP arrays using different SNP resources have been developed using appropriate platforms. For example, in rice, RiceSNP6K [9], RiceSNP50K [10] and HDRA700K [11] were developed based on the Illumina Infinium platform; 1536 SNPs [12] based on the GoldenGate platform; 384-ples [13] based on the BeadXpress platform; and GeneChip Rice 44 K [14] and OsSNPnks 50 K chip [15] were developed using the Affymetrix platform. In cotton, only one array (CottonSNP63K) has been developed based on the Illumina Infinium platform [16]. The array loci were identified from 13 different discovery sets that represent a diverse range of *G. hirsutum* germplasms, as well as five other species. Using the array, 188 RIL populations from an intra-specific cross (HS46 and MARCABUCAG8US-1-88) were genotyped, and 71 QTLs for fiber quality and yield traits were detected [17]. Recently, GWAS analysis of 719 diverse accessions of upland cotton was conducted using multiple environment tests of fiber quality traits with the CottonSNP63K SNP array, and 10,511 polymorphic SNPs and forty-six significant SNPs associated with five fiber quality traits were detected [18]. In addition, the population structure and genetic basis of the agronomic traits of upland cotton has been investigated in 503 *G. hirsutum* accessions using 11,975 quantified polymorphic SNPs in the CottonSNP63K SNP array [19]. These results indicate that the SNP array is a robust and highly efficient tool for use in cotton genetic studies and breeding improvement.

The availability of data on the whole-genome of four different cotton species, two diploid cotton species, *G. raimondii* and *G. arboreum* [20, 21], the upland cotton genetic standard line, *G. hirsutum* acc. TM-1 [22, 23], and two sea-island cottons, *G. barbadense* cv. Xinhai21 and *G. barbadense* acc. 3–79 [24, 25], made it possible to develop SNP markers at a genome-wide level. In our previous studies, an ultra-dense inter-specific genetic map of *G. hirsutum* and *G. barbadense* containing 519 framework SSRs and 4,999,048 SNPs, was constructed [26]. Further, genomic re-sequencing data from 100 different upland cotton cultivars were produced and provided millions of polymorphic SNP information [27]. In this study, based on the high quality reference sequence of the TM-1 genome [22] and re-sequencing data from different upland cotton cultivars [27], we focused on the development of a high-density upland cotton SNP array for intraspecific genotyping detection, with the advantages on addressable SNPs, genome-wide distribution, high-effective genotyping detection. We selected 82,259 SNPs for the array development using Illumina’s Infinium platform, and named this the CottonSNP80K. Our tests showed that this array had wide applicability, good repeatability and high efficiency of genotyping identification. Phylogenetic relationship and GWAS analysis of salt stress traits indicated the CottonSNP80K play important roles in germplasm genotyping, variety verification, genetic relationship identification, functional genomics studies, and molecular breeding in cotton.

## Results

### Development and characteristics of the CottonSNP80K array

**Fig. 1:**
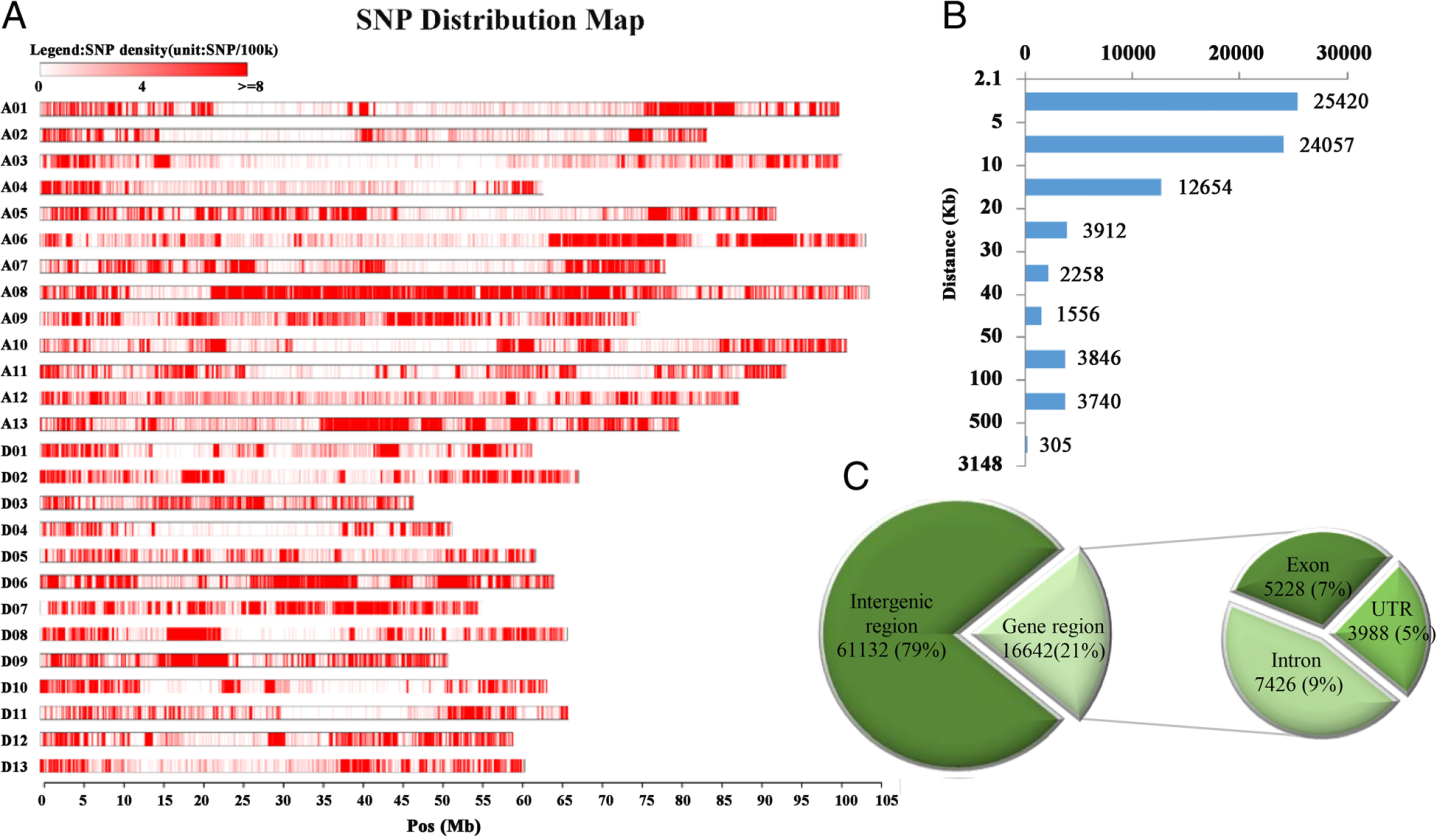
Description of SNPs in CottonSNP80K array. **a** SNPs distributions on the 26 chromosomes of upland cotton. A01-A13 and D01-D13 in vertical axis are the serial number of 26 chromosomes; the horizontal axis shows chromosome length (Mb); the red region depicts SNP density (the number of SNPs per window). **b** Distances between the SNPs. The vertical axis represents distances range (Kb) of SNPs. **c** Distribution of genic and intergenic regions of selected SNPs

Based on the high quality reference sequence of the TM-1 genome [22] and re-sequencing data from 100 different cultivars of *G. hirsutum* with an average coverage of 5× in the genome [27], we developed a high-density SNP array for high-throughput intraspecific upland cotton genotyping identification. Following the SNP selection process (Additional file 1: Figure S1), 82,259 SNPs were selected out of 1,372,195 putative intraspecific SNPs with minor allele frequencies (MAFs) >0.1, and 77,774 of these (94.55%) were successfully synthesized on the array. We named the array CottonSNP80K. In this array, the average distance between adjacent SNPs was 24.9 Kb (Fig. 1a). In detail, 45,183 (58.10%) and 32,591 loci (41.90%) were located in the At- and Dt- subgenomes, and one SNP existed every 25.7 Kb and 23.8Kb, respectively. The highest SNP density was on A08, which contained 7773 SNPs (~1 SNP every 13.3 Kb), followed by chromosomes D06, D07, D09 and A13 (the number of SNPs ranged from 2938 to 4340, with an average of 1 SNP every 15–20 Kb). Chromosomes A02, A03, A04, and D04 had the relatively low SNP density, with an average distance of more than 40 Kb between two adjacent SNPs (Table 1). The distances between the loci of 63.62% (49,477) of the SNPs were less than 10 Kb, and only 7891 SNPs (10.15%) had gaps that were >50 Kb. The largest gap between two adjacent SNPs was 3148 Kb, and this was on chromosome A03 (Fig. 1b). In CottonSNP80K, 16,642 SNPs (21.40%) were tagged in the genic region of 9902 genes, and 61,132 SNPs (78.60%) in intergenic regions. Of the genic region SNPs, 44.62% (7426), 31.42% (5228) and 23.96% (3988) were distributed within introns, exons and untranslated regions (UTRs), respectively (Fig. 1c).

**Table 1:** Chromosome distribution of SNP loci in CottonSNP80K array in TM-1 genome

Chr.	No. of SNP	Chromosome length (Kb)	Average distance (Kb)	Chr.	No. of SNP	Chromosome length (Kb)	Average distance (Kb)
A01	3500	99,884.70	28.5	D01	2339	61,456.01	26.3
A02	1996	83,447.91	41.8	D02	2985	67,284.55	22.5
A03	2466	100,263.05	40.7	D03	1889	46,690.66	24.7
A04	1434	62,913.77	43.9	D04	1272	51,454.13	40.5
A05	3384	92,047.02	27.2	D05	2041	61,933.05	30.3
A06	4698	103,170.44	22.0	D06	4037	64,294.64	15.9
A07	3070	78,251.02	25.5	D07	3472	55,312.61	15.9
A08	7773	103,626.34	13.3	D08	2898	65,894.14	22.7
A09	3621	74,999.93	20.7	D09	2938	50,995.44	17.4
A10	2964	100,866.60	34.0	D10	2130	63,374.67	29.8
A11	2897	93,316.19	32.2	D11	1866	66,087.77	35.4
A12	3040	87,484.87	28.8	D12	2562	59,109.84	23.1
A13	4340	79,961.12	18.4	D13	2162	60,534.30	28.0
At	45,183	1,160,232.96	25.7	Dt	32,591	774,421.80	23.8
Total	77,774	1,934,654.76	24.9				

### SNP genotype calling in allotetraploid cotton

The genome-wide CottonSNP80K array was used to genotype 352 cotton cultivars/accessions (Additional file 2: Table S1). GenomeStudio Genotyping software (V2011.1, Illumina, Inc.) was used to provide a default clustering file based on bi-allelic SNPs, and all 77,774 SNP markers corresponding to the three separated signal clusters, AA, AB and BB, were genotyped. From an evolutionary point of view, the polyploidy cotton originated from an interspecific hybridization event between A- and D-genome diploid species about 1–2 million years ago, and the two extant progenitor relatives diverged from a common ancestor 5–10 million years ago [28]. In addition, upland cotton is a type of cross-pollination allotetraploid crop with a 10–15% natural hybridization rate. Based on this, some SNP loci in upland cotton could contain five genotypes (AAAA, AAAB, AABB, ABBB and BBBB). When these genotyping signals gather >3 clusters, automatic SNP calling might make mistakes, therefore we confirmed these loci genotypes with the manual adjustment.

**Fig. 2:**
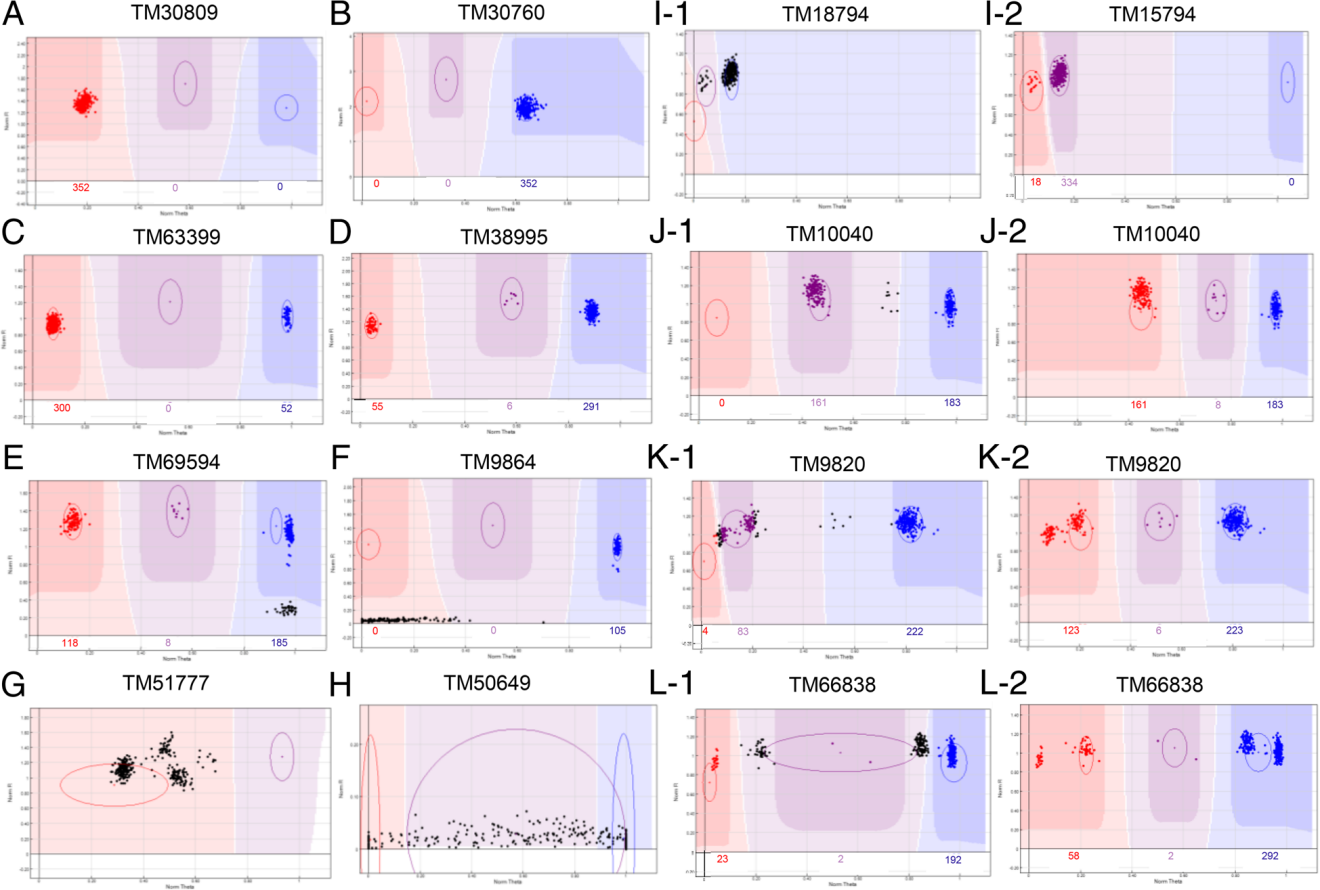
The typical cluster graph of SNP markers in CottonSNP80K array. **a**-**d** bi-allelic SNPs, can be accurately recognize by GenomeStudio software. **e** CNV, noted as “NG”; **f** InDel, noted as “--”. **g**-**h** Complex cluster graph difficult to group accurately and as missing data. **i**-**l** Corrected SNPs, 1 and 2 indicated default clustering using GenomeStudio software and adjusted clustering, respectively

We grouped the SNP cluster graphs of 352 samples into three types. In the first type, the loci of most samples could be accurately recognized with default clustering by GenomeStudio software. Markers of this type primarily produced four distinct clustering patterns. Those with the first and second patterns (Fig. 2a, b) all fell in a single cluster, representing a monomorphic locus. Those with the third and fourth patterns (Fig. 2c, d) were markers that showed two (AA, BB) and three (AA, AB, AB) clearly definable clusters, respectively, and were homozygous genotype clusters located near 0 and 1. The second type of SNP cluster graph consisted of markers that had partial or whole samples with uncalled genotypes. The partial uncalled markers may be caused by structural variance (SV) or insertion-deletion (In-Del), and were noted as “NG” rather than “--” (Fig. 2e, f). In addition, data for 165 SNPs was missing from all samples, because these SNPs showed complex cluster graphs that could not be accurately clustered even with manual adjustment or the NormR >0.2 in these SNPs (Fig. 2g, h). In the third type, most loci needed to be adjusted for accurate genotyping (Fig. 2i-2l). Some monomorphic loci that were relatively close together by GenomeStudio genotyping were adjusted to produce two cluster groups (AA and AB), of which the only possible genotypes were AAAA and AAAB in allotetraploid cotton (Fig. 2i ). By adjusting cluster AB to AA, the heterozygous rate significantly reduced for some loci with high heterozygous rates, of which the possible genotypes were AABB, ABBB and BBBB (Fig. 2j). A small number of loci showed 4–5 distinct clusters (Fig. 2k, l): their genotypes might be AAAA, AAAB, AABB, ABBB or BBBB, in which two closer groups (such as AAAA and AAAB or ABBB and BBBB) would be merged into one. Through the adjustment, a more accurate clustering file was produced to improve the genotyping efficiency of the 352 cotton samples.

The genotype data revealed that the 352 tested accessions possessed an average call rate of 99.23%. Of the 77,774 SNPs in the array, 77,252 (99.33%) had high call rates >95% in these tested accessions. Of them, 59,502 SNPs (76.51%) showed polymorphic loci, with MAFs > 0.05 for 57,071 loci (95.91%) and MAFs > 0.1 for 48,940 (82.25%), respectively. Compared to the Yellow River Valley and the introduced accessions, the Yangtze River Valley and Northwestern inland region accessions had more SNPs with MAFs > 0.1 (Table 2). The number of polymorphic SNPs was very similar in the Yangtze River Valley (59,428) and the Yellow River Valley accessions (59,402), however, the number of SNPs with MAFs > 0.1 was higher in the Yangtze River Valley accessions (46,434) than in the Yellow River Valley accessions (40,540), indicating the higher genetic diversity in the accessions from the Yangtze River Valley than that in the Yellow River Valley. We also found that the number of rare alleles (those with a frequency of less than 5%) in the Yellow River Valley accessions was greater than that in the Yangtze River Valley accessions.

**Table 2:** Summary of the SNP polymorphisms detected in the different cotton subpopulations^a^

Origin	Accession No.	Accessions with phenotype data	Polymorphic SNPs	SNPs MAF > 0.05	SNPs MAF > 0.1
The Yellow River Valley	182	166	59,402	49,975	40,540
The Yangtze River Valley	79	75	59,428	55,337	46,434
Northwestern inland region	22	22	57,294	51,875	46,656
Northern specifically early maturation region	16	16	56,085	53,888	45,126
The introduced landraces	13	9	52,113	49,884	40,745
Outgroup	20	0	54,310	51,404	43,336
Total	332	288	59,502	57,071	48,940

^a^MAF, minor allele frequency; SNP, single nucleotide polymorphism

Accessions with phenotype data for GWAS analysis between SNP loci and salt-tolerance related traits

Outgroup was involved in 5 wild cotton, 13 semi-wild cotton and 2 sea-island cotton materials

### Evaluation of the CottonSNP80K array

To evaluate the genotyping accuracy, reproducibility, and distinguishability of the CottonSNP80K array, 22 cotton materials were selected for genotyping analysis (Additional file 2: Table S1). First of all, we analyzed the SNPs of TM-1 and Hai7124 by comparing their array detection with the re-sequencing results from genomic data of 62× TM-1 and 39× Hai7124 [26]. The SNPs that could be detected by both methods were used for comparison, and the consistency of the two SNP calling results was 95.40% for TM-1 and 97.19% for Hai7124.

To determine the reproducibility of this array, *G. hirsutum* acc. W0 DNA samples were loaded three times on different chips and exhibited 100% identical genotype calls. Two biological replicates of TM-1 plants from different seed sources also exhibited 99.99% consistency. Further, the consistencies of two biological replicates of Zhongmiansuo12, the immature fiber mutant and Xuzhou-142 were 94.96%, 90.06% and 93.00%, respectively. In addition, interspecific polymorphism between *G. hirsutum* and *G. barbadense* was also detected; with the polymorphic rate of 32.70% between TM-1 and Hai7124, and 32.85% between TM-1 and Junhai1 (Table 3). Taken together, these results show that the CottonSNP80K array has a very good genotyping efficiency and accuracy.

**Table 3:** Polymorphism rate and the similarity analysis using CottonSNP80K array

Materials	Polymorphic rate (%)	Materials	Similarity (%)
TM-1 vs Hai7124	32.70	Three W0 DNA samples	100.00
TM-1 vs Junhai1	32.85	Two TM-1 biological replicates	99.99
7235 vs 7235 mutant	25.01	Two Xuzhou 142 biological replicates	93.00
Xinxiangxiaoji linted-fuzzless vs Xinxiangxiaoji lintless-fuzzless	16.11	Two Zhongmiansuo12 biological replicates	94.96
Xuzhou 142 vs Xuzhou 142 lintless-fuzzless mutant	19.04	Two *im* mutant biological replicates	90.06
TM-1 vs SL1–7-1	26.82		
TM-1 vs MD-17	30.62		
TM-1 vs N1	22.73		
TM-1 vs n2	25.71		
TM-1 vs *im* mutant	21.00		

To detect the distinguishability of this array, the polymorphic SNP loci of several mutants and their corresponding donors with similar genetic backgrounds were mined (Table 3). The near-isogenic lines Xinxiangxiaoji linted-fuzzless and Xinxiangxiaoji lintless-fuzzless yielded a 16.11% polymorphic rate. The polymorphic rates were 19.04% between Xuzhou-142 and the Xuzhou-142 lintless-fuzzless mutant, and 25.01% between 7235 and the 7235 mutant. We also detected relatively high polymorphic rates between TM-1 and four fiber development mutants (SL1–7-1, MD-17, N1, and n2), which ranged from 22.73% to 30.62%. In addition, a 21.00% polymorphic rate was identified between TM-1 and the immature fiber (*im*) mutant, far higher than the 1.28% polymorphic rate from SSR markers reported previously [29]. In general, the CottonSNP80K array showed high distinguishability when genotyping upland cotton accessions with similar genetic backgrounds.

**Fig. 3:**
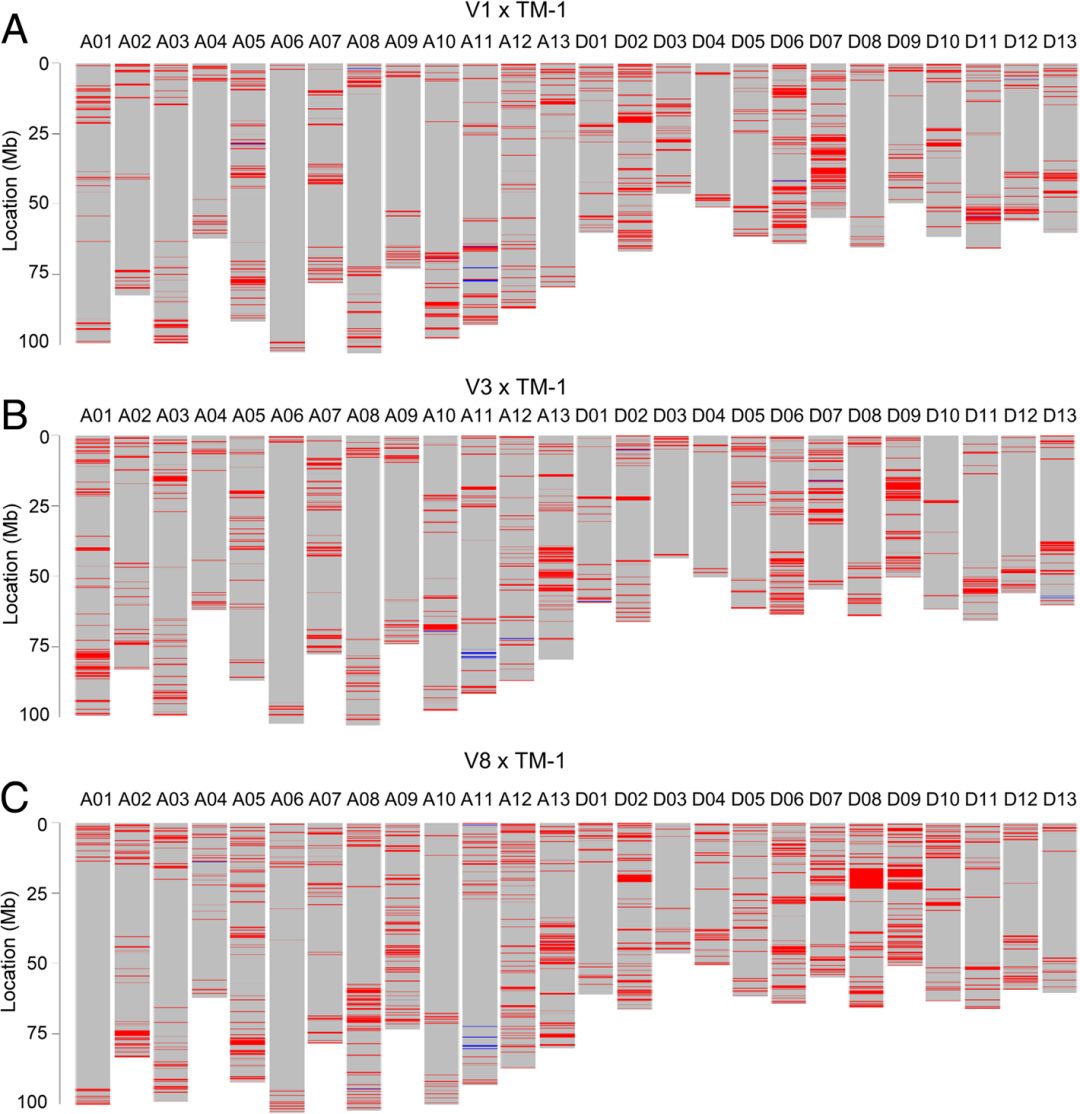
Verification of heterozygous loci in three F_1_ combinations. **a** (V1 × TM-1) F_1_; **b** (V3 × TM-1) F_1_; **c** (V8 × TM-1) F_1_. The red labels on the chromosomes displayed the expected heterozygous loci which showed different homozygous alleles in two parents. Otherwise labeled in blue

To confirm the accuracy of the heterozygous loci, we compared genotype data from three parent/F_1_ combinations: (V1 × TM-1), (V3 × TM-1), and (V8 × TM-1). The results showed that the heterozygous rate increased by 14.07–19.91% in F_1_, compared with the corresponding parents. In the parents, 99.02%, 98.55% and 99.11% of the polymorphic SNP loci showed perfect heterozygous loci in the corresponding F_1_ (Fig. 3), indicating the potential application of the array in the identification of different varieties and hybrids.

### Phylogenetic tree and linkage disequilibrium analysis of upland cotton core collections

**Fig. 4:**
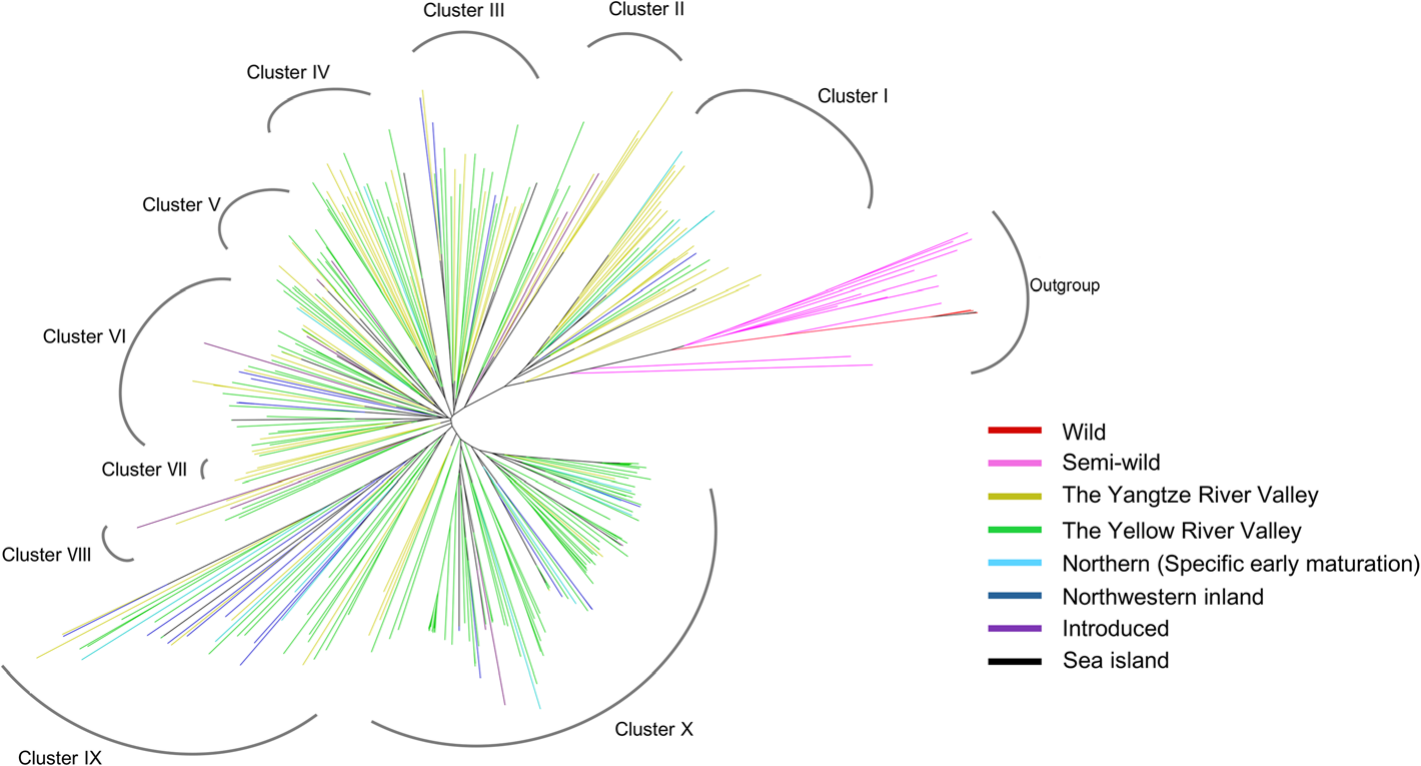
Phylogenetic analysis of 332 cotton accessions based on the CottonSNP80K genotyping array. A neighbor-joining tree was constructed using 57,071 polymorphic SNP markers

**Fig. 5:**
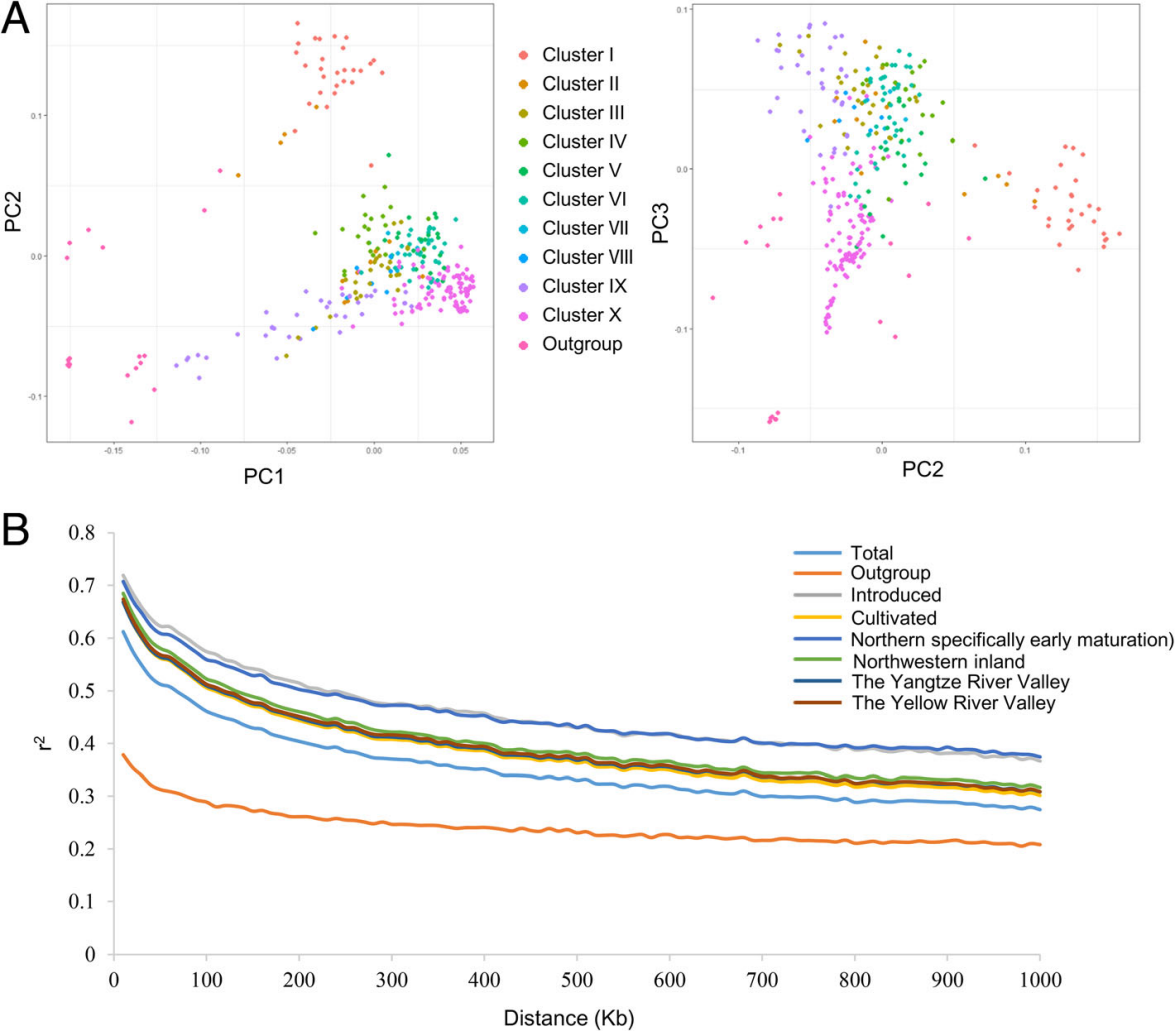
PCA and linkage disequilibrium analysis of the 332 cotton accessions. **a** PCA for the 332 cotton accessions based on CottonSNP80K genotyping data. **b** LD decay of r^2^ and physical distance between SNP markers in different cotton groups. The 332 cotton accessions were classified into the introduced landraces (named as Introduced), the Chinese modern improved cultivars respectively from the Yellow River Valley, the Yangtze River Valley, the Northwestern inland region and the Northern specifically early maturation region (named as Cultivated), and the outgroup (named as Outgroup). Samples from the same group are represented by the same color

Phylogenetic analysis and principal component analysis (PCA) were used to compare the genetic diversity between wild cotton, semi-wild cotton and upland cotton accessions. A neighbor-joining tree was constructed using 57,071 polymorphic SNP markers with MAFs >0.05 (Fig. 4). As a result, the 332 samples were clustered into two groups. One contained 20 accessions of wild, semi-wild and sea-island cotton clustered into one branch as outgroup. The other contained 312 *G. hirsutum* accessions, including 299 from modern improved Chinese cultivars/accessions and 13 from the introduced landraces. We further classified the group into ten clusters, clusterI to clusterX. We found that the classification of 299 upland cotton accessions from four different ecological areas (the Yellow River Valley, the Yangtze River Valley, the Northwestern inland region and the Northern specifically early maturation region), was irrelevant to their geographical distribution, but highly related to the introduced landraces. Several landraces, from the United States and other countries, were assigned to different groups, respectively. For example: Uganda3, Stoneville4, DPL16, King, Stoneville2B, DPL15, and Foster6 were grouped into clustersI-VI andX, respectively, indicating that these landraces have high genetic diversity and contributed to the development of different breeding populations in China during the improvement of modern varieties. Further, PCA analysis also agreed well with the clustering results in the phylogenetic tree (Fig. 5a).

Linkage disequilibrium (LD) analysis was performed for cotton accessions of different origins using PLINK software. Pairwise LD was estimated using squared allele frequency correlations (R^2^). The results showed that the distance of LD decay in the outgroup was shorter than in other groups, implying that cultivated upland cotton, especially that from the northern group, may have been under higher selective pressure during evolution. Further, the accessions from the Yangtze River Valley, the Yellow River Valley and the Northwestern inland groups showed the same LD patterns, indicating the narrow genetic diversity of modern improved cultivars. On average, the distance of LD decay in the 332 cotton accessions was ~700 K (Fig. 5b).

### Application in genome-wide association studies for salt stress traits

**Fig. 6:**
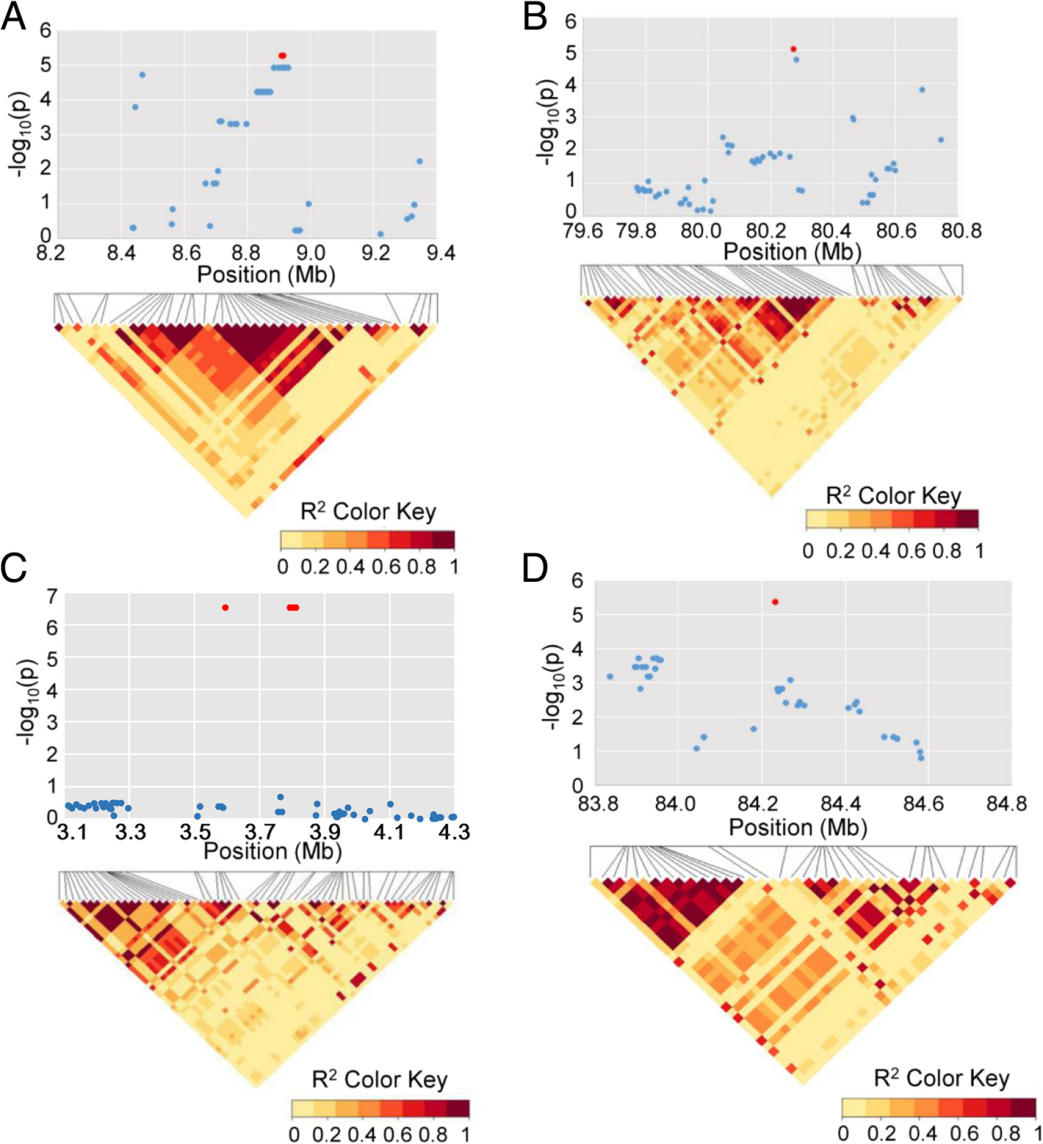
GWAS analysis for salt-tolerance related traits in cotton. Local Manhattan plot (top) and LD heatmap (bottom) surrounding the peak of candidate loci. The significant SNPs (*P* < 1 × 10^−5^) were marked in red. The pair-wise LD between the SNP markers is indicated as D’ values, where dark red indicated a value of 1 and light yellow indicated 0. **a** SNPs associated with relative chlorophyll content (RCC) at the peak region (8.41–9.41 Mb) on chromosome D05. **b** SNPs associated with relative MDA content (RMDA) at the peak region (79.77–80.77 Mb) on chromosome A02. **c** SNPs associated with relative MDA content (RMDA) at the peak region (3.10–4.31 Mb) on chromosome D09. **d** SNPs associated with relative germination rate (RGR) at the peak region (83.73–84.73 Mb) on chromosome A12

Based on previous investigations of 10 salt stress traits that provided phenotype data for 288 *G. hirsutum* accessions [30] (Additional file 3: Table S2), GWAS analysis was performed using a total of 54,588 SNPs (MAFs > 0.05) and stress traits. Eight significant SNPs for three salt stress traits were detected at the *P* < 1 × 10^−5^ level (Fig. 6). Among those, two loci located on D5 (TM57102 and TM57104), were significantly associated with relative chlorophyll content (RCC); one locus located on A2 (TM5633) and four loci located on D9 (TM70162, TM70169, TM70170, TM70171) were significantly associated with relative MDA content (RMDA); and one locus located on A12 (TM43002) was significantly associated with relative germination rate (RGR) (Table 4). Considering the LD decay distance of ~700 K identified in this study, we further analyzed the sequence information in 500 Kb regions flanking each peak SNP, and tagged candidate genes in the four significant SNP regions associated with salt stress traits. One LD block was associated with RCC on chromosome D05 (8.41–9.41 Mb) and RGR on A12 (83.73–84.73 Mb), and two LD blocks were associated with RMDA on chromosomes A02 (79.77–80.77 Mb) and D09 (3.10–4.31 Mb) (Fig. 6). A total of 308 genes were annotated in the four different regions (Additional file 4: Table S3), and 36 and 21 genes were annotated as response to stimulus and stress, respectively (Additional file 5: Table S4).

**Table 4:** Information on eight SNPs associated with salt-tolerance related traits based on GWAS analysis

Traits^a^	SNPs	Chr.	Position (bp)	Allele	MAF	-Log_10_(P)	Allele's effect
RCC	TM57102	D5	8,908,622	A/G	0.36(G)	5.23	0.06
	TM57104	D5	8,918,010	A/C	0.14(C)	5.23	0.06
RMDA	TM5633	A2	80,274,022	A/G	0.18(A)	5.00	−0.28
	TM70162	D9	3,595,148	A/G	0.47(A)	6.53	−0.13
	TM70169	D9	3,793,378	A/C	0.48(A)	6.53	−0.13
	TM70170	D9	3,801,945	A/G	0.47(A)	6.53	−0.13
	TM70171	D9	3,812,331	A/T	0.47(T)	6.53	−0.13
RGR	TM43002	A12	84,233,507	A/G	0.17(G)	5.34	0.13

^a^ *RCC* relative chlorophyll content, *RMDA* relative MDA content, *RGR* relative germination rate

## Discussion

### Development of CottonSNP80K

High-density SNP arrays have been developed for a number of economically important crops, such as rice [9–11], maize [31, 32], soybean [33], wheat [34], and cotton [16]. These chips have been successfully used for functional genomics studies and molecular breeding. To date, only one array (CottonSNP63K) has been reported in cotton [16]. In this study, we developed a new upland cotton genotyping SNP array (CottonSNP80K), an improved version of CottonSNP63K for upland cotton intraspecific genotyping detection. Of the 82,259 SNPs selected, 77,774 SNPs (94.55%) were successfully synthesized on this CottonSNP80K array. Compared to the CottonSNP63K array [16], the CottonSNP80K array shows several significant highlights and improvements. Firstly, compared to SNP loci in CottonSNP63K array, which were collected from 13 different discovery sets of *G. hirsutum* germplasm and five other species, the SNP loci in CottonSNP80K array benefited from the whole genome sequencing of *G. hirsutum* acc. TM-1 [22]*,* and 1,372,195 intraspecific non-unique SNPs identified by re-sequencing of *G. hirsutum* accessions [27], therefore the selected SNPs in CottonSNP80K could be distributed along the entire genome. Secondly, the CottonSNP63K array contains 63,058 markers, including 45,104 intraspecific SNPs and 17,954 interspecific SNPs, whereas the CottonSNP80K array increased the total number of markers to 77,774. With requirement of MAFs > 0.1 by analyzing the re-sequencing data of different cotton accessions, the SNPs in CottonSNP80K showed five to six times upland cotton intraspecific polymorphism compared with that in CottonSNP63K. In the recent reports, using the CottonSNP63K array, Huang et al. (2017) [19] detected 11,975 quantified polymorphic SNPs in a diverse and nationwide population containing 503 *G. hirsutum* accessions, and Sun et al. (2017) [18] detected 10,511 polymorphic SNPs using 719 diverse accessions of upland cotton. In the present study, the number of polymorphic markers for upland cotton intraspecific genotyping detection was increased to 59,502 using the CottonSNP80K array. Thirdly, compared with the CottonSNP63K array, each SNP marker in the CottonSNP80K array is addressable, which avoids the disturbance of homeologous/paralogous genes. During the development of the CottonSNP80K array, we also considered factors affecting the array quality, including flanking sequence information, Illumina design scores, heterozygosity rates, cluster results, which ensures that it is of high quality in upland cotton genotyping detection.

Upland cotton accounts for more than 90% of the world’s cotton production, however, modern upland cotton cultivars have narrow genetic diversity. The CottonSNP80K array is more suitable for upland cotton intraspecific genotyping detection, which can greatly overcome the narrow genetic background and low genetic diversity. Using the CottonSNP80K array, genotyping analysis was performed on 352 cotton accessions. Of the 77,774 SNPs on the array, 59,502 (76.51%) were polymorphic loci, with 95.91% (57,071) and 82.25% (48,940) showing MAFs greater than 0.05 and 0.1, respectively. In the CottonSNP63K project, these parameters were much lower, at 66.8% (MAF > 0.05) and 55.8% (MAF > 0.1) [16]. We also investigated the genetic diversity of upland cotton accessions from different ecological areas. The Yangtze River Valley and the Yellow River Valley showed a higher degree of polymorphism than Northwestern inland and Northern groups. In addition, the number of SNPs with MAF > 0.1 was higher in the Yangtze River accessions than in the Yellow River Valley accessions, indicating that more rare alleles exist in the Yellow River Valley group. We also found that the rate of polymorphisms between *G. hirsutum* and *G. barbadense* was greater than 30%, implying the array has potential for interspecific genotyping analysis.

To evaluate the reproducibility and the distinguishability of the CottonSNP80K array, three parent/F_1_ combinations, several mutants and their corresponding donors with similar genetic backgrounds, and the replicated cotton varieties were used for genotyping analysis. In the replication analysis, technical DNA replicates showed perfect consistency (100%) and different levels of variability were detected with biological replication types. Considerable genetic variation existed in cotton lines of different origins, which might be related to the cross-pollination nature of upland cotton with a 10–15% natural hybridization rate. Similarly, this variation was also found in other array projects [16, 35]. Thus, the CottonSNP80K array provides an efficient tool to characterize the inconsistencies between upland cotton accessions despite their similar genetic backgrounds. In addition, the CottonSNP80K array presented an excellent ability to detect heterozygous loci with an accuracy of more than 98%. Taken together, these findings suggest that the CottonSNP80K array is of high quality with high levels of convenience and cost-effectiveness, and could be widely used in diverse types of research.

### Applications of CottonSNP80K

Cotton (*Gossypium* spp.) is the world’s most important fiber crop plant. While most of the >50 *Gossypium* species are diploid (*n* = 13), five are allopolyploids (*n* = 26), originating from an interspecific hybridization event between A- and D-genome diploid species [36]. There are three wild tetraploid cotton species (*G. tomentosum*, *G. mustelinum* and *G. darwinii*) and two cultivated species, upland cotton (*G. hirsutum*) and sea-island cotton (*G. barbadense*). In addition, upland cotton consists of seven semi-wild races, *G. hirsutum* race *punctatum*, *morrilli*, *yucatanense*, *richmondii*, *marie-galante*, *latifolium* and *palmeri*, and the domesticated upland cotton cultivars, which constitute about 90% of the world cotton production. In this study, using the CottonSNP80K array, 5, 13 and 312 cotton accessions from wild species, *G. hirsutum* races and cultivated upland cotton accessions, respectively, as well as 2 sea-island cotton accessions, were selected for phylogenetic analysis. We found that 20 accessions from wild, semi-wild and sea-island cotton were clustered together, with closer genetic relationships between 2 sea-island cotton accessions and wild species, and more similarity between semi-wild species and cultivated upland cotton. This might be because these SNP loci originated from intraspecific upland cotton variation. We also found that all 312 *G. hirsutum* accessions were sub-clustered ten groups, but these groupings were not related to their geographical distribution. A similar analysis was reported in a previous study that used 81,675 SNPs from the SLAF-seq of 355 cotton accessions, and population structure analysis showed that the tested accessions could be separated into nine subpopulations with no obvious geographic relationship [8]. In the present study, we grouped 312 *G. hirsutum* accessions, including 299 from modern improved Chinese cultivars/accessions and 13 from the introduced landraces, into ten clusters, clusterI to clusterX. We found that the different introduced landraces were clustered in different subgroups, such as King (in the 1920s), Foster6 (in 1933), Stoneville2B (in 1947), DPL15 (in 1950) and DPL16 (in 1970), which were successively introduced into China from the USA and were grouped into clusters IV, X, V, VI and III, respectively. In addition, Stoneville 4, which was introduced from the USA in 1934 was grouped into cluster II. Uganda3, which was introduced from Uganda in 1959, was grouped into clusterI. Junmian1 was developed from the filial generation of multi-parents involved in C1470, C3521, and 147φ, which was introduced from the former Soviet Union in 1960s, and was grouped to cluster IX. These results suggest that the introduced upland cotton landraces have a high genetic diversity, which provides rich genetic resources for breeding modern improved cotton varieties in China.

Soil salinization, one of the main factors leading to soil desertification and land degradation, has become a serious threat to agricultural production and ecological environments throughout the world. More than 800 million hectares (~6%) of world’s total land area are salt affected [37]. Compared with several other crops such as rice, maize and soybean, cotton is a pioneer crop in saline-alkali land. Based on SSR markers, some of the QTLs related to salt tolerance traits have been reported by family-based QTL mapping [38, 39] and association mapping approaches [30, 40–42]. To date, no studies have reported the identification of genes/QTLs associated with salinity tolerance in cotton using high-density SNP arrays. In previous studies, we carried out the large-scale identification of ten salt tolerance related traits in 304 upland cotton cultivars/accessions [30]. Here, we integrated 288 cotton accessions with genome-wide SNP genotyping data, and a total of 54,588 SNPs (MAF > 0.05) were used for GWAS analysis of salt stress related traits. We detected eight significant SNPs for three salt stress traits at the *P* < 1 × 10^−5^ level (Table 4, Fig. 6). Further, in these SNPs peak region, 36 and 21 genes were annotated as response to stimulus and response to stress, respectively. Of these candidate stress response genes, mitogen-activated protein kinase kinase 5 (MKK5), which acts upstream of MPK3/MPK6, plays key roles in mediating many different stress signals and in plant development [43]. Overexpression of MKK5 in wild-type plants enhanced the tolerance to salt treatments, while mkk5 mutants exhibited hypersensitivity to salt stress during germination on salt-containing media in *Arabidopsis* [44]. Another candidate gene, lesion simulating disease 1 (LSD1), together with phytoalexin deficient 4 (PAD4) and enhanced disease susceptibility 1 (EDS1), comprise a molecular hub that integrates plant responses to several stresses such as hypoxia [45], drought [46], cold [47] and oxidative stress [48]. A reactive oxygen species (ROS) scavenging related gene, peroxidase (POD), was also found amongst the candidate genes. ROS, typically in the forms of H_2_O_2_ and O^2−^, can be rapidly generated in plants when exposed to adverse environments, such as high salinity, drought, heat or cold. An excess of ROS leads to oxidative damage of cellular components, such as proteins, lipids, carbohydrates and nucleic acids. High ROS level also disturb protein synthesis and the cellular membrane, resulting in cellular and tissue damage [49]. Therefore, ROS scavenging capability is closely related to plant stress tolerance. Transcription factors (TFs) are considered as upstream regulatory proteins that play a major role in cellular metabolism and abiotic stress responses. One TF, named homeobox 7 (HB7), which encodes a putative transcription factor that contains a homeodomain closely linked to a leucine zipper motif, was found in an associated region with relative germination rate (RGR) in this study. ATHB7 has essential functions in the primary response to drought, as mediators of a negative feedback effect on ABA signaling in the plant response to water deficit [50]. Independent expression of AtHB7 resulted in improved stress tolerance in *Arabidopsis* [51]. In summary, with the identification of increasing numbers of genes/QTL related to important traits, high-throughput genotyping platforms will provide an effective pathway for high-resolution dissection of complex traits and molecular breeding by design in cotton.

With reduced computational requirements for downstream data processing, high call frequency, low error rates and ease of use, high-density SNP arrays are an attractive genotyping tool, which are widely applied in diversity studies and high-resolution dissection of complex traits [34], variety verification, trait introgression [10], and genome-wide association studies [18]. Compared with genome-wide sequencing technology, the SNP loci in the array is known and addressable, the data generation and analysis are more convenient and cost-effective. In the study, CottonSNP80K showed to work efficiently for variety verification and genome-wide association studies for salt stress traits, indicating great application potential in future cotton molecular breeding.

## Conclusion

Based on reference sequence of the TM-1 genome and re-sequencing data from 100 different cultivars of *G. hirsutum*, high-density SNP arrays of CottonSNP80K was developed. By genotyping 352 cotton cultivars/accessions, the CottonSNP80K showed excellent efficiency with an average call rate of 99.23%. Application tests using cotton accessions with parent/F_1_ combinations or with similar genetic backgrounds showed that this array had high genotyping accuracy, good repeatability, and wide applicability. Phylogenetic relationship and GWAS analysis of salt stress traits showed that the CottonSNP80K played important roles in variety verification, genetic relationship identification, and molecular breeding in cotton. Compared to the previously reported CottonSNP63K array, the CottonSNP80K array showed higher SNP density with genome-wide distribution, more accurate addressable loci, and higher upland cotton intraspecific polymorphism, which will be a powerful tool for genetics and molecular breeding by design in cotton.

## Methods

### Plant materials

A total of 352 cotton materials were collected for the evaluation and application of the CottonSNP80K array in this study (Additional file 2: Table S1). To evaluate the CottonSNP80K array, 22 cotton materials (named as E1-E22 in Additional file 2: Table S1), comprising the sequenced reference lines (*G. hirsutum* acc. TM-1 and *G. barbadense* cv. Hai 7124), duplicated DNA samples, parent/F_1_ combinations, several mutants and their corresponding donors with similar genetic background, *G. hirsutum* cv. Zhongmiansuo12 and *G. barbadense* cv. Junhai 1, were genotyped. To investigate the genetic diversity and phylogenetic relationship, a core collection of 332 cotton accessions were chosen (named as C1-C332 in Additional file 2: Table S1), including 5 wild and 13 semi-wild *G. hirsutum* race materials, 312 cultivated upland cotton accessions and two sea-island cotton accessions. For the GWAS analysis, 288 *G. hirsutum* accessions (selected from C21-C332 in Additional file 2: Table S1) with salt-stress phenotyping identified in our previous study [27] (Additional file 3: Table S2) were chosen for marker-trait associations of salt stress traits.

### Selection of SNPs and development of the cotton SNP array

To develop the genome-wide CottonSNP80K chip, an Illumina Infinium array, as well as intraspecific SNPs data from sequencing of the allotetraploid cotton *G. hirsutum* acc. TM-1 [22] and re-sequencing of 100 different cultivars in *G. hirsutum* with 5× coverage on average [27] were used. In total, 1,372,195 putative intraspecific SNPs with MAF > 0.1 were detected and chosen for inclusion on the array. When designing the array, subsequent filtering steps included the following: (1) genotype accuracy was required to be >99.12%; (2) SNPs in repeat regions were filtered; (3) no other SNPs or InDels were permitted in the 50 bp flanking the SNP site; (4) heterozygosity rates were required to be <15%; (5) SNP cluster analysis was carried out. After these filters were applied, 175,192 SNPs remained and were submitted through the Illumina Assay Design Tool to determine array design scores for each marker. SNPs in gene regions with Illumina design scores >0.7, and SNPs in intergenic regions with Illumina design scores >0.9 remained. Further, the inter-marker distance flanking the SNPs was >2100 bp. The remaining 82,259 SNP markers were used for the manufacture of the CottonSNP80K array by Illumina (Additional file 6: Table S5). The scheme of CottonSNP80K development is shown in Additional file 1: Figure S1.

### Array hybridization and SNP genotyping

Genomic DNA from young leaf tissue of the 352 cotton materials involved in this study was isolated as described by Paterson et al. (1993) [52]. According to the Illumina protocols, qualified DNA was hybridized to the CottonSNP80K array. The Illumina iScan array scanner was used to scan arrays, and GenomeStudio Genotyping software (V2011.1, Illumina, Inc.) was used to cluster SNP alleles and for genotyping. Among the 82,259 SNP markers, the CottonSNP80K array successfully synthesized 77,774 SNP markers (94.55%). That is, 4485 SNPs failed to meet bead representation thresholds and were removed. At first, the default clustering file was provided based on bi-allelic SNPs. Next, all 77,774 SNPs were tested and manually adjusted as described by Hulse-Kemp et al., (2015b) [16]. Ultimately, an adjusted clustering file was produced and was used to call SNP genotypes for the allotetraploid cottons in the study.

### Phylogenetic trees and linkage disequilibrium analysis

PLINK V1.90 software [53] was employed to conduct similarity analysis of 332 cotton accessions for cluster analysis. Based on the distance matrix data (1-IBS, identity-by-state), phylogenetic trees were constructed using neighbor software, and visually edited by figtree software. Principal component analysis (PCA) was performed using an IBS matrix data. The correlation coefficient (r^2^) of alleles was calculated to measure LD in each upland cotton group level using PLINK V1.90, and LD blocks containing SNP loci associated with target traits were generated using the R software package “LDheatmap”.

### GWAS analysis and identification of candidate genes

A total of 54,588 SNPs (MAF > 0.05) were used for the GWAS analysis of 288 upland cotton accessions. We carried out GWAS analysis with TASSEL 5.0, which uses a Mixed Linear Model (MLM) with Q + K model for association tests [54]. Population structure (Q) was performed using admixture 1.3 [55] with k = 3, and kinship matrix (K) was performed with Tassel 5.0. Significant levels of association were estimated considering an adjusted *P* value of 1/n (1.8 × 10^−5^) after the Bonferroni correction, which n represented the number of SNP markers. To get more reliable results, the SNPs with *P* values lower than 1 × 10^−5^ were selected as finally significant trait-associated SNPs. Candidate genes were identified within 500 Kb upstream or downstream of peak SNPs (the most significant SNPs). Manhattan plots were performed using the R software package “qqman”. GO analysis was implemented using AgriGO, and genes in “response to stimulate” or “response to stress” terms were selected as candidate genes.

## Additional files

Additional file 1: Figure S1. The flow chart of developing the CottonSNP80K array. (TIFF 229 kb)

Additional file 2: Table S1. Basic information and their phylogenetic classification on cotton materials in this study. (XLSX 25 kb)

Additional file 3: Table S2. Phenotype data of 10 salt-stress traits in the 288 tested cotton accessions. (XLSX 53 kb)

Additional file 4: Table S3. Gene information of SNPs associated with salt-tolerance related traits within 1 Mb regions. (XLSX 31 kb)

Additional file 5: Table S4. Information on candidate genes response to stimulus and stress. (XLSX 12 kb)

Additional file 6: Table S5. Information on SNP loci in the CottonSNP80K array. (XLSX 9317 kb)

## Abbreviations

EDS1: enhanced disease susceptibility 1
GBS: genotyping-by-sequencing
GWAS: genome-wide association studies
HB7: homeobox 7
In-Del: insertion-deletion
LD: Linkage disequilibrium
LSD1: lesion simulating disease 1
MAFs: minor allele frequencies
MAGIC: multi-parent advanced generation inter-cross
MAS: marker-assisted selection
MKK: mitogen-activated protein kinase kinase
PAD4: phytoalexin deficient 4
PCA: principal component analysis
POD: peroxidase
QTL: quantitative trait loci
RCC: relative chlorophyll content
RGR: relative germination rate
RILs: recombination inbreed lines
RMDA: relative malondialdehvde content
ROS: reactive oxygen species
SLAF-seq: specific-locus amplified fragment sequencing
SNP: single nucleotide polymorphism
SSR: simple sequence repeat
SV: structural variation
TFs: transcription factors
UTRs: untranslated regions

## References

[1] Bowman DT, May OL, Calhoun DS. Genetic base of upland cotton cultivars released between 1970 and 1990. Crop Sci. 1996;36(3):577–81.

[2] Wang Q, Fang L, Chen JD, Hu Y, Si ZF, Wang S, Chang LJ (2015). Genome-wide mining, characterization, and development of microsatellite markers in *Gossypium* species. Sci Rep.

[3] Deschamps S, Llaca V, May GD (2012). Genotyping-by-sequencing in plants. Biology.

[4] Logan-Young CJ, Yu JZ, Verma SK, Percy RG, Pepper AE (2015). SNP discovery in complex allotetraploid genomes (*Gossypium* Spp., Malvaceae) using genotyping by sequencing. Appl Plant Sci.

[5] Hulse-Kemp AM, Ashrafi H, Stoffel K, Zheng X, Saski CA, Scheffler BE, Fang DD (2015). BAC-end sequence-based SNP mining in allotetraploid cotton (*Gossypium*) utilizing resequencing data, phylogenetic inferences, and perspectives for genetic mapping. Genes Genomes Genetics.

[6] Islam MS, Thyssen GN, Jenkins JN, Zeng L, Delhom CD, McCarty JC, Deng DD (2016). A MAGIC population-based genome-wide association study reveals functional association of GhRBB1_A07 gene with superior fiber quality in cotton. BMC Genomics.

[7] Su JJ, Fan SL, Li LB, Wei HL, Wang CX, Wang HT, Song MZ (2016). Detection of favorable qtl alleles and candidate genes for lint percentage by gwas in chinese upland cotton. Front Plant Sci.

[8] Su JJ, Pang CY, Wei HL, Li LB, Liang B, Wang CX, Song MZ (2016). Identification of favorable SNP alleles and candidate genes for traits related to early maturity via GWAS in upland cotton. BMC Genomics.

[9] Yu HH, Xie WB, Li J, Zhou FS, Zhang QF (2014). A whole-genome SNP array (RICE6K) for genomic breeding in RICE. Plant Biotechnol J.

[10] Chen H, Xie W, He H, Yu H, Chen W, Li J, Yu R (2014). A high-density SNP genotyping array for rice biology and molecular breeding. Mol Plant.

[11] McCouch SR, Wright MH, Tung CW, Maron LG, McNally KL, Fitzgerald M, Singh N (2016). Open access resources for genome-wide association mapping in rice. Nat Commun.

[12] Zhao K, Wright M, Kimball J, Eizenga G, McClung A, Kovach M, Tyagi W (2010). Genomic diversity and introgression in O sativa reveal the impact of domestication and breeding on the rice genome. PLoS One.

[13] Chen H, He H, Zou Y, Chen W, Yu R, Liu X, Yang Y (2011). Development and application of a set of breeder-friendly SNP markers for genetic analyses and molecular breeding of rice (Oryza Sativa L.). Theor Appl Genet.

[14] Zhao K, Tung CW, Eizenga GC, Wright MH, Ali ML, Price AH, Norton GJ (2011). Genome-wide association mapping reveals a rich genetic architecture of complex traits in *Oryza sativa*. Nat Commun.

[15] Singh N, Jayaswal PK, Panda K, Mandal P, Kumar V, Singh B, Mishra S (2015). Single-copy gene based 50 K SNP chip for genetic studies and molecular breeding in rice. Sci Rep.

[16] Hulse-Kemp AM, Lemm J, Plieske J, Ashrafi H, Buyyarapu R, Fang DD, Frelichowski J (2015). Development of a 63K SNP array for cotton and high-density mapping of intraspecific and interspecific populations of *Gossypium* spp. Genes Genomes Genetics.

[17] Li C, Dong Y, Zhao T, Li L, Li C, Yu E, Mei L (2016). Genome-wide SNP linkage mapping and QTL analysis for fiber quality and yield traits in the upland cotton recombinant inbred lines population. Front Plant Sci.

[18] Sun Z, Wang X, Liu Z, Gu Q, Zhang Y, Li Z, Ke H, et al. Genome-wide association study discovered genetic variation and candidate genes of fibre quality traits in *Gossypium hirsutum* L. Plant Biotechnol J. 2017; doi 10.1111/pbi.12693. 10.1111/pbi.12693 PMC5506648 28064470

[19] Huang C, Nie X, Shen C, You C, Li W, Zhao W, Zhang X, et al. Population structure and genetic basis of the agronomic traits of upland cotton in China revealed by a genome-wide association study using high-density SNPs. Plant Biotechnol J. 2017; doi:10.1111/pbi.12722. 10.1111/pbi.12722 PMC5633765 28301713

[20] Paterson AH, Wendel JF, Gundlach H, Guo H, Jenkins J, Jin DC, Llewellyn D (2012). Repeated polyploidization of *Gossypium* genomes and the evolution of spinnable cotton fibres. Nature.

[21] Li F, Fan G, Wang K, Sun F, Yuan Y, Song G, Li Q (2014). Genome sequence of the cultivated cotton *Gossypium arboreum*. Nat Genet.

[22] Zhang T, Hu Y, Jiang W, Fang L, Guan X, Chen J, Zhang J (2015). Sequencing of allotetraploid cotton (Gossypium Hirsutum L. acc. TM-1) provides a resource for fiber improvement. Nat Biotechnol.

[23] Li FG, Fan GY, Lu CR, Xiao GH, Zou CS, Kohel RJ, Ma ZY (2015). Genome sequence of cultivated upland cotton (*Gossypium hirsutum* TM-1) provides insights into genome evolution. Nat Biotechnol.

[24] Liu X, Zhao B, Zheng HJ, Hu Y, Lu G, Yang CQ, Chen JD (2015). *Gossypium barbadense* genome sequence provides insight into the evolution of extra-long staple fiber and specialized metabolites. Sci Rep.

[25] Yuan DJ, Tang ZH, Wang MJ, Gao WH, Tu LL, Jin X, Chen LL (2015). The genome sequence of Sea-Island cotton (*Gossypium barbadense*) provides insights into the allopolyploidization and development of superior spinnable fibres. Sci Rep.

[26] Wang S, Chen JD, Zhang WP, Hu Y, Chang LJ, Fang L, Wang Q (2015). Sequence-based ultra-dense genetic and physical maps reveal structural variations of allopolyploid cotton genomes. Genome Biol.

[27] Fang L, Gong H, Hu Y, Liu C, Zhou B, Huang T, Wang Y (2017). Genomic insights into divergence and dual domestication of cultivated allotetraploid cottons. Genome Biol.

[28] Wendel JF, Cronn RC (2003). Polyploidy and the evolutionary history of cotton. Adv Agron.

[29] Wang C, Zhang TZ, Guo WZ (2012). The *im* mutant gene negatively affects many aspects of fiber quality traits and lint percentage in cotton. Crop Sci.

[30] Du L, Cai C, Wu S, Zhang F, Hou S, Guo W (2016). Evaluation and exploration of favorable QTL alleles for salt stress related traits in cotton cultivars (*G. hirsutum* L.). PLoS One.

[31] Ganal MW, Durstewitz G, Polley A, Berard A, Buckler ES, Charcosset A, Clarke JD (2011). A large maize (*Zea mays* L.) SNP genotyping array: development and germplasm genotyping and genetic mapping to compare with the B73 reference genome. PLoS One.

[32] Unterseer S, Bauer E, Haberer G, Seidel M, Knaak C, Ouzunova M, Meitinger T (2014). A powerful tool for genome analysis in maize: development and evaluation of the high density 600 k SNP genotyping array. BMC Genomics.

[33] Song Q, Hyten DL, Jia G, Quigley CV, Fickus EW, Nelson RL, Cregan PB (2013). Development and evaluation of SoySNP50K, a high-density genotyping array for soybean. PLoS One.

[34] Wang S, Wong D, Forrest K, Allen A, Chao S, Huang BE, Maccaferri M (2014). Characterization of polyploid wheat genomic diversity using a high-density 90000 single nucleotide polymorphism array. Plant Biotechnol J.

[35] Yan JB, Shah T, Warburton ML, Buckler ES, McMullen MD, Crouch J (2009). Genetic characterization and linkage disequilibrium estimation of a global maize collection using SNP markers. PLoS One.

[36] Wendel JF (1989). New world tetraploid cottons contain old world cytoplasm. Proc Natl Acad Sci U S A.

[37] Munns R, Tester M (2008). Mechanisms of salinity tolerance. Annu Rev Plant Biol.

[38] Tiwari RS, Picchioni GA, Steiner RL, Jones DC, Hughs SE, Zhang JF (2013). Genetic variation in salt tolerance at the seedling stage in an interspecific backcross inbred line population of cultivated tetraploid cotton. Euphytica.

[39] Oluoch G, Zheng JY, Wang XX, Khan MKR, Zhou ZL, Cai XY, Wang CY (2016). QTL mapping for salt tolerance at seedling stage in the interspecific cross of *Gossypium tomentosum* with *Gossypium hirsutum*. Euphytica.

[40] Jia YH, Sun JL, Wang XW, Zhou ZL, Pan ZE, He SP, Pang BY (2014). Molecular diversity and association analysis of drought and salt tolerance in *Gossypium hirsutum* L. germplasm. J Integr Agric.

[41] Saeed M, Guo W, Zhang T (2014). Association mapping for salinity tolerance in cotton (*Gossypium hirsutum* L) germplasm from US and diverse regions of China. Austrilian J Crop Sci.

[42] Zhao YL, Wang HM, Shao BX, Chen W, Guo ZJ, Gong HY, Sang XH, et al. SSR-based association mapping of salt tolerance in cotton (*Gossypium hirsutum* L). Genet Mol Res. 2016;15(2) doi:10.4238/gmr.15027370. 10.4238/gmr.15027370 27323090

[43] Andreasson E, Ellis B (2010). Convergence and specificity in the *Arabidopsis* MAPK nexus. Trends Plant Sci.

[44] Xing Y, Chen WH, Jia W, Zhang J (2015). Mitogen-activated protein kinase kinase 5 (MKK5)-mediated signalling cascade regulates expression of iron superoxide dismutase gene in Arabidopsis under salinity stress. J Exp Bot.

[45] Muhlenbock P, Plaszczyca M, Plaszczyca M, Mellerowicz E, Karpinski S (2007). Lysigenous aerenchyma formation in Arabidopsis is controlled by LESION SIMULATING DISEASE1. Plant Cell.

[46] Szechynska-Hebda M, Czarnocka W, Hebda M, Bernacki MJ, Karpinski S (2016). PAD4, LSD1 and EDS1 regulate drought tolerance plant biomass production and cell wall properties. Plant Cell Rep.

[47] Huang X, Li Y, Zhang X, Zuo J, Yang S (2010). The *Arabidopsis* LSD1 gene plays an important role in the regulation of low temperature-dependent cell death. New Phytol.

[48] Wituszynska W, Szechynska-Hebda M, Sobczak M, Rusaczonek A, Kozlowska-Makulska A, Witon D, Karpinski S (2015). Lesion simulating disease 1 and enhanced disease susceptibility 1 differentially regulate UV-C-induced photooxidative stress signaling and programmed cell death in *Arabidopsis thaliana*. Plant Cell Environ.

[49] Wojcik KA, Kaminska A, Blasiak J, Szaflik J, Szaflik JP (2013). Oxidative stress in the pathogenesis of keratoconus and Fuchs endothelial corneal dystrophy. Int J Mol Sci.

[50] Valdes AE, Overnas E, Johansson H, Rada-Iglesias A, Engstrom P (2012). The homeodomain-leucine zipper (HD-zip) class I transcription factors ATHB7 and ATHB12 modulate abscisic acid signalling by regulating protein phosphatase 2C and abscisic acid receptor gene activities. Plant Mol Biol.

[51] Lee YH, Chun JY (1998). A new homeodomain-leucine zipper gene from *Arabidopsis thaliana* induced by water stress and abscisic acid treatment. Plant Mol Biol.

[52] Paterson AH, Brubaker C, Wendel JF (1993). A rapid method for extraction of cotton (*Gossypium* spp) genomic DNA suitable for RFLP or PCR analysis. Plant Mol Biol Rep.

[53] Purcell S, Neale B, Todd-Brown K, Thomas L, Ferreira MAR, Bender D, Maller J (2007). PLINK: a tool set for whole-genome association and population-based linkage analyses. Am J Hum Genet.

[54] Bradbury PJ, Zhang Z, Kroon DE, Casstevens TM, Ramdoss Y, Buckler ES (2007). TASSEL: software for association mapping of complex traits in diverse samples. Bioinformatics.

[55] Alexander DH, Novembre J, Lange K (2009). Fast model-based estimation of ancestry in unrelated individuals. Genome Res.

