# Supplementary figures and images for: High-density 80 K SNP array is a powerful tool for genotyping G. hirsutum accessions and genome analysis
Source: BMC Genomics. 2017 Aug 23;18:654. doi: 10.1186/s12864-017-4062-2 (PMC5569476; doi:10.1186/s12864-017-4062-2)

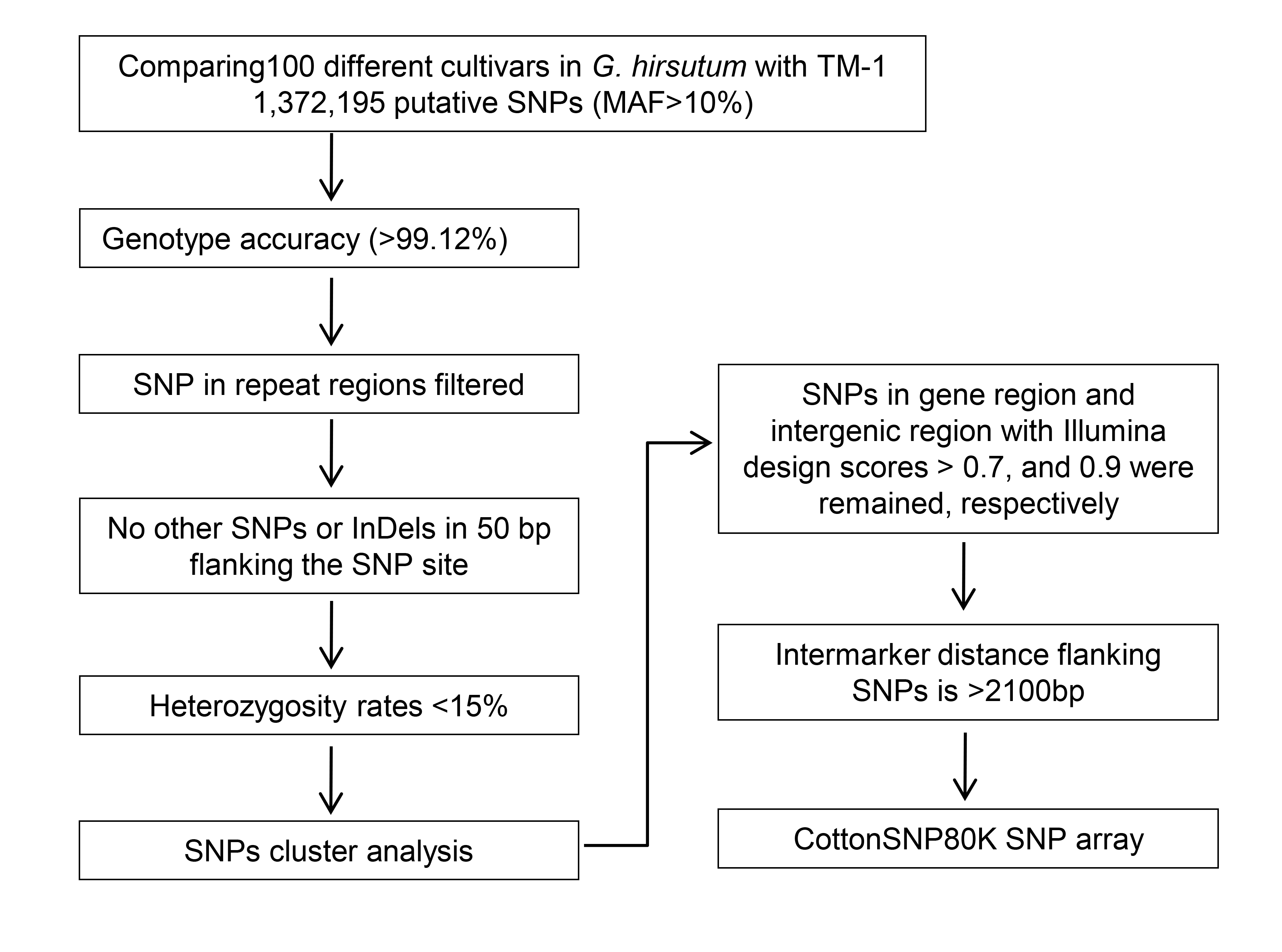

Supplement: Supplementary file 1 — The flow chart of developing the CottonSNP80K array. (TIFF 229 kb) [file 12864_2017_4062_MOESM1_ESM.tif]
